# Pre-clinical dose-ranging efficacy, pharmacokinetics, tissue biodistribution, and toxicity of a targeted contrast agent for MRI of amyloid deposition in Alzheimer’s disease

**DOI:** 10.1038/s41598-020-73233-7

**Published:** 2020-09-30

**Authors:** Andrew A. Badachhape, Peter K. Working, Mayank Srivastava, Prajwal Bhandari, Igor V. Stupin, Laxman Devkota, Eric A. Tanifum, Ananth V. Annapragada, Ketan B. Ghaghada

**Affiliations:** 1 Department of Radiology, Baylor College of Medicine, Houston, TX 77030 USA; 2 Alzeca Biosciences Inc., Houston, TX 77057 USA; 3 The Singleton Department of Pediatric Radiology, Texas Children’s Hospital, Houston, TX 77030 USA; 4 Department of Radiology, Baylor College of Medicine, 1102 Bates Street, Suite 850, Houston, TX 77030 USA; 5 Department of Pediatrics-Oncology, Baylor College of Medicine, Houston, TX 77030 USA; 6 Edward B. Singleton Department of Radiology, Texas Children’s Hospital, Houston, USA

**Keywords:** Alzheimer's disease, Nanoparticles, Magnetic resonance imaging, Molecular imaging

## Abstract

In these preclinical studies, we describe **ADx-001**, an Aβ-targeted liposomal macrocyclic gadolinium (Gd) imaging agent, for MRI of amyloid plaques. The targeting moiety is a novel lipid-PEG conjugated styryl-pyrimidine. An MRI-based contrast agent such as **ADx-001** is attractive because of the lack of radioactivity, ease of distribution, long shelf life, and the prevalence of MRI scanners. Dose-ranging efficacy studies were performed on a 1 T MRI scanner using a transgenic APP/PSEN1 mouse model of Alzheimer’s disease. **ADx-001** was tested at 0.10, 0.15, and 0.20 mmol Gd/kg. Gold standard post-mortem amyloid immunostaining was used for the determination of sensitivity and specificity. **ADx-001** toxicity was evaluated in rats and monkeys at doses up to 0.30 mmol Gd/kg. **ADx-001** pharmacokinetics were determined in monkeys and its tissue distribution was evaluated in rats. **ADx-001**-enhanced MRI demonstrated significantly higher (p < 0.05) brain signal enhancement in transgenic mice relative to wild type mice at all dose levels. **ADx-001** demonstrated high sensitivity at 0.20 and 0.15 mmol Gd/kg and excellent specificity at all dose levels for in vivo imaging of β amyloid plaques. **ADx-001** was well tolerated in rats and monkeys and exhibited the slow clearance from circulation and tissue biodistribution typical of PEGylated nanoparticles.

## Introduction

A definitive diagnosis of Alzheimer’s disease (AD) requires postmortem neuropathological demonstration of β amyloid plaques and neurofibrillary tau tangles. However, advances in the development of position emission tomography (PET) imaging probes for these biomarkers have facilitated a new ‘research’ framework’ to study and characterize the disease in vivo^1^. Although not approved for clinical diagnosis, this research framework, advanced by the National Institute of Aging and Alzheimer’s Association (NIA-AA), characterizes biological AD by either in vivo PET imaging or other biomarker evidence of β amyloid plaques and neurofibrillary tau tangles. The use of targeted PET tracers in clinical research has improved our understanding of the evolution of AD biomarkers in the context of dementia^2–6^. It is expected that the clinical deployment of non-invasive imaging AD biomarkers will enable early diagnosis of AD-related dementia and facilitate early intervention.

The build-up of β amyloid plaques in the brain is one of the earliest pathological events in AD^7–10^. Pre-clinical and clinical studies using PET probes have demonstrated that parenchymal deposition of amyloid plaques begins decades before clinical presentation of cognitive impairment in AD-related dementia^11–14^. Furthermore, the formation of amyloid plaques has been causally linked to the pathogenesis of neurofibrillary tau tangles^7,15,16^. Although amyloid imaging PET probes, such as [^18^F]-florbetaben, [^18^F]-florbetapir and [^18^F]-flutemetamol, have substantially advanced our understanding of AD pathophysiology leading to cognitive impairment and are playing a critical role in clinical trials for the evaluation of disease-modifying investigational therapies, access to PET for the general population remains a worldwide problem. An amyloid imaging agent for use with magnetic resonance imaging (MRI) could be transformative due to ease of access, substantially lower cost, and the prevalence of MRI in current AD patient workup.

**Figure 1:**
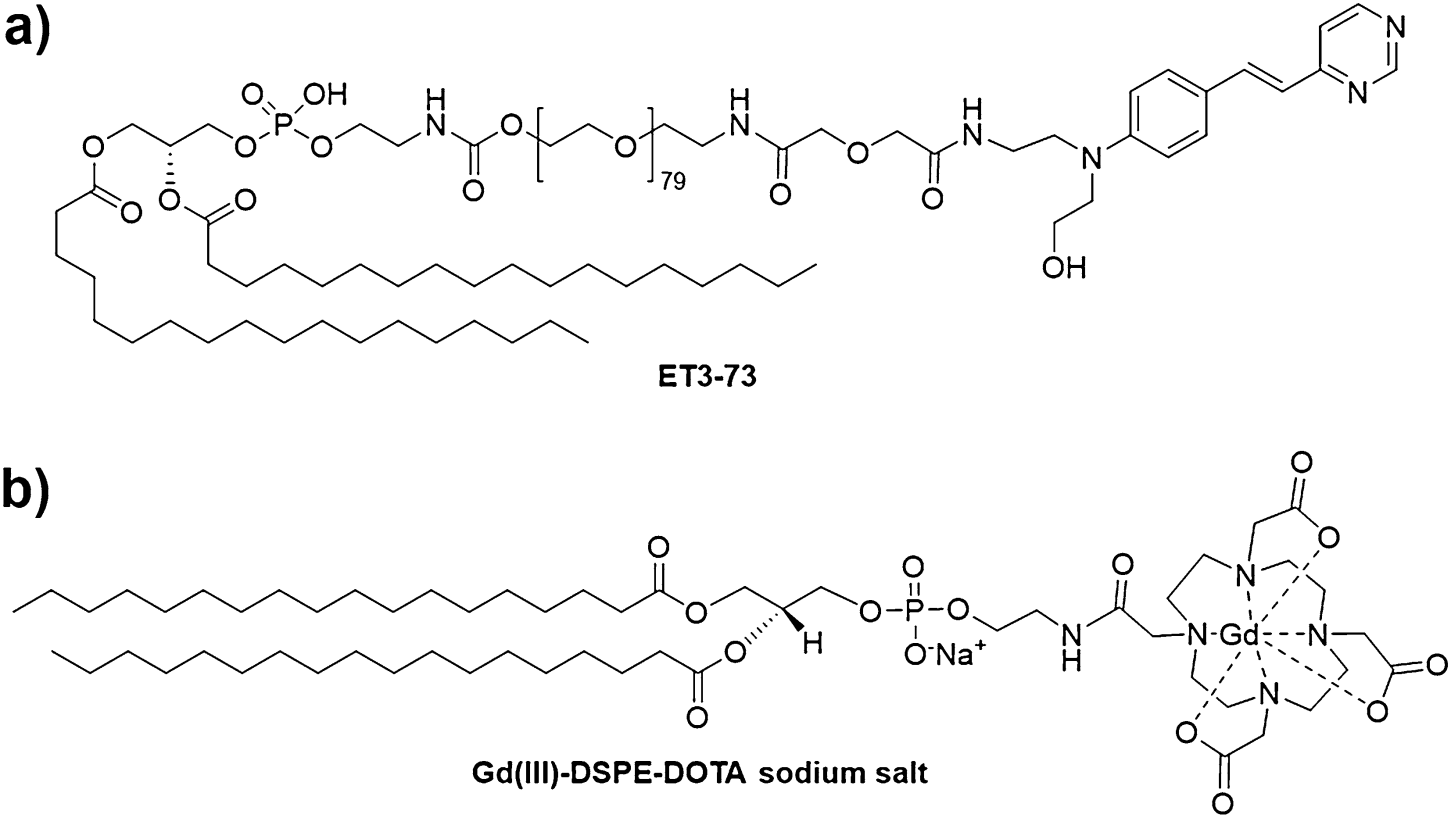
Key components in the formulation of ADx-001. (**a**) Structure of amyloid plaque targeting molecule (ET3-73). (**b**) Structure of macrocyclic gadolinium imaging molecule (Gd(III)-DSPE-DOTA sodium salt).

Previous pre-clinical studies have demonstrated that a high T1 relaxivity, amyloid-targeted liposomal-gadolinium (Gd) nanoparticle contrast agent (containing a linear Gd chelate, Gd-DTPA) enables in vivo MR imaging of amyloid plaques in transgenic mouse models of AD^17^. Following emerging evidence of brain deposition of Gd dissociated from such linear chelates^18^, an agent using the same targeting ligand (Fig. 1a), but based on a macrocyclic DOTA chelator (Fig. 1b) was developed. In this work, we describe results of pre-clinical toxicity, pharmacokinetics, biodistribution, and dose-ranging efficacy studies of this targeted liposomal macrocyclic Gd contrast agent, referred to as **ADx-001**, for MR imaging of amyloid plaques.

## Results

### In vivo MRI

**Figure 2:**
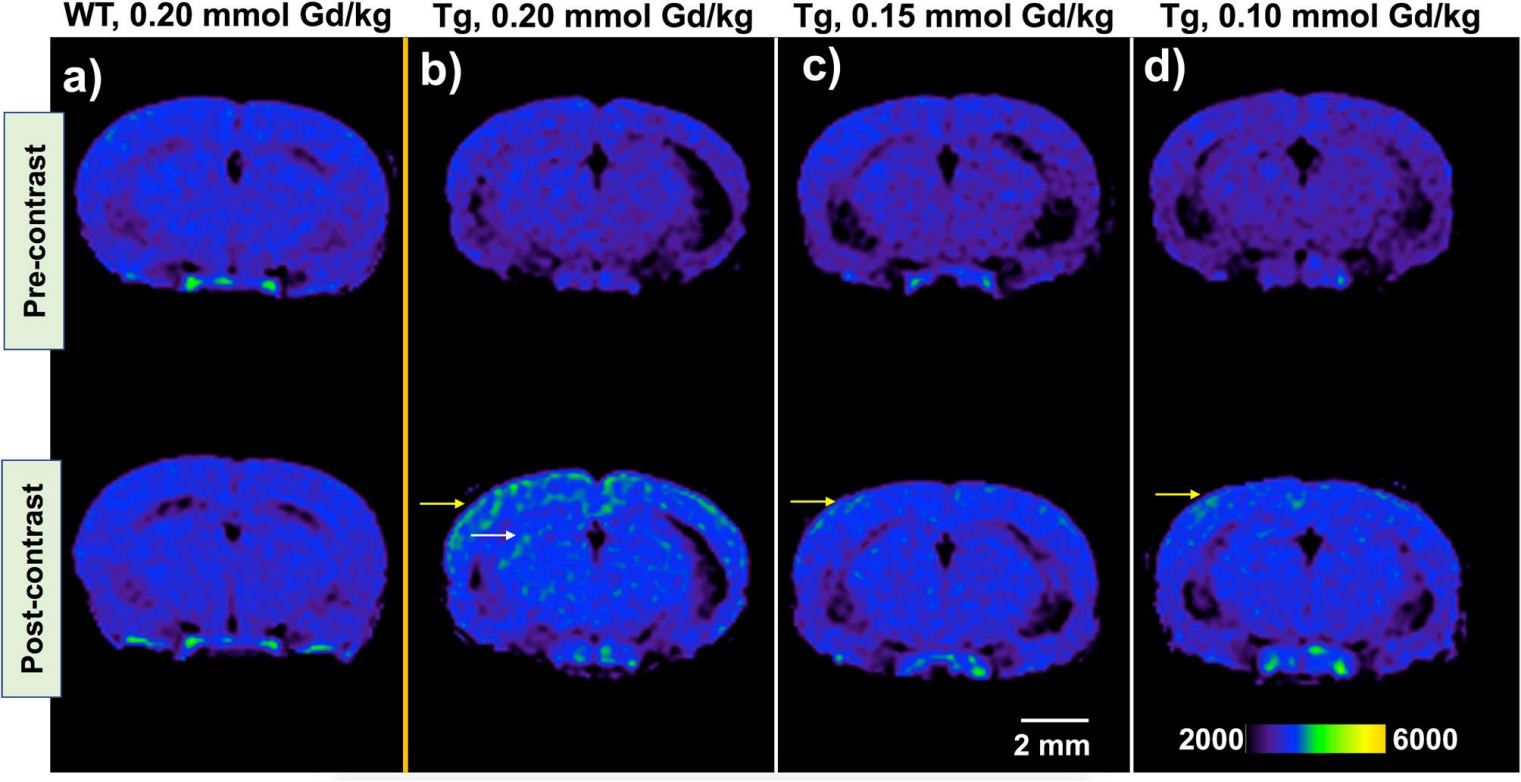
T1-weighted spin-echo (T1w-SE) images demonstrate signal enhancement in delayed post scans of transgenic (Tg) APPswe/PSEN1dE9 mice but not in age-matched, wild type (WT) mice. (**a**) WT mouse administered 0.2 mmol Gd/kg of **ADx-001** shows little to no signal enhancement four days after injection. (**b**) Transgenic mouse shows high enhancement in cortical (yellow arrow) and hippocampal regions (white arrow) four days after administration of 0.2 mmol Gd/kg of **ADx-001**. (**c**) Transgenic mouse shows low enhancement in cortical region (yellow arrow) four days after administration of 0.15 mmol Gd/kg of **ADx-001**. (**d**) Transgenic mouse shows low enhancement in cortical region (yellow arrow) four days after administration of 0.10 mmol Gd/kg of ADx-001. Scale bar represents 2 mm. All images are windowed to same signal intensity color bar scale.

**Figure 3:**
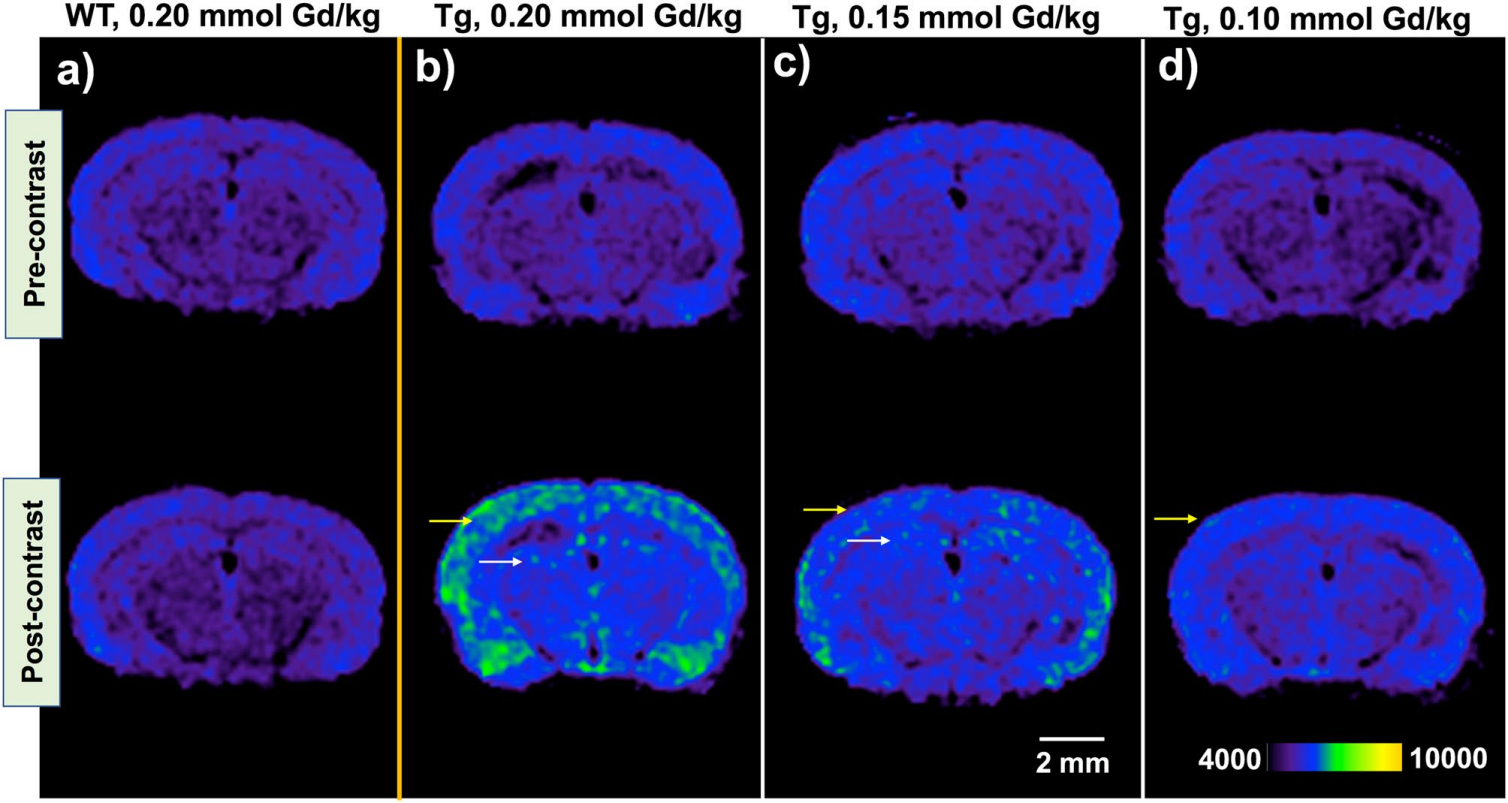
Fast spin-echo inversion recovery (FSE-IR) axial images demonstrate MR signal enhancement in **ADx-001** delayed post-contrast scans of transgenic (Tg) APPswe/PSEN1dE9 mice, but not in age-matched, wild type (WT) control mice. (**a**) WT mouse administered 0.20 mmol Gd/kg **ADx-001** demonstrates no signal enhancement in delayed post-contrast images. (**b**) Transgenic mouse administered 0.20 mmol Gd/kg **ADx-001** shows high signal enhancement in cortical (yellow arrow) and hippocampal regions (white arrow) in delayed post-contrast images. (**c**) Transgenic mouse administered 0.15 mmol Gd/kg **ADx-001** shows moderate signal enhancement in cortical region (yellow arrow) and low enhancement in hippocampal region (white arrow) in delayed post-contrast images. (**d**) Transgenic mouse administered 0.10 mmol Gd/kg **ADx-001** shows low signal enhancement in cortical region (yellow arrow) in delayed post-contrast images. All delayed post-contrast images were acquired four days after administration of **ADx-001**. Scale bar represents 2 mm. All images are windowed to same signal intensity color bar scale.

**Figure 4:**
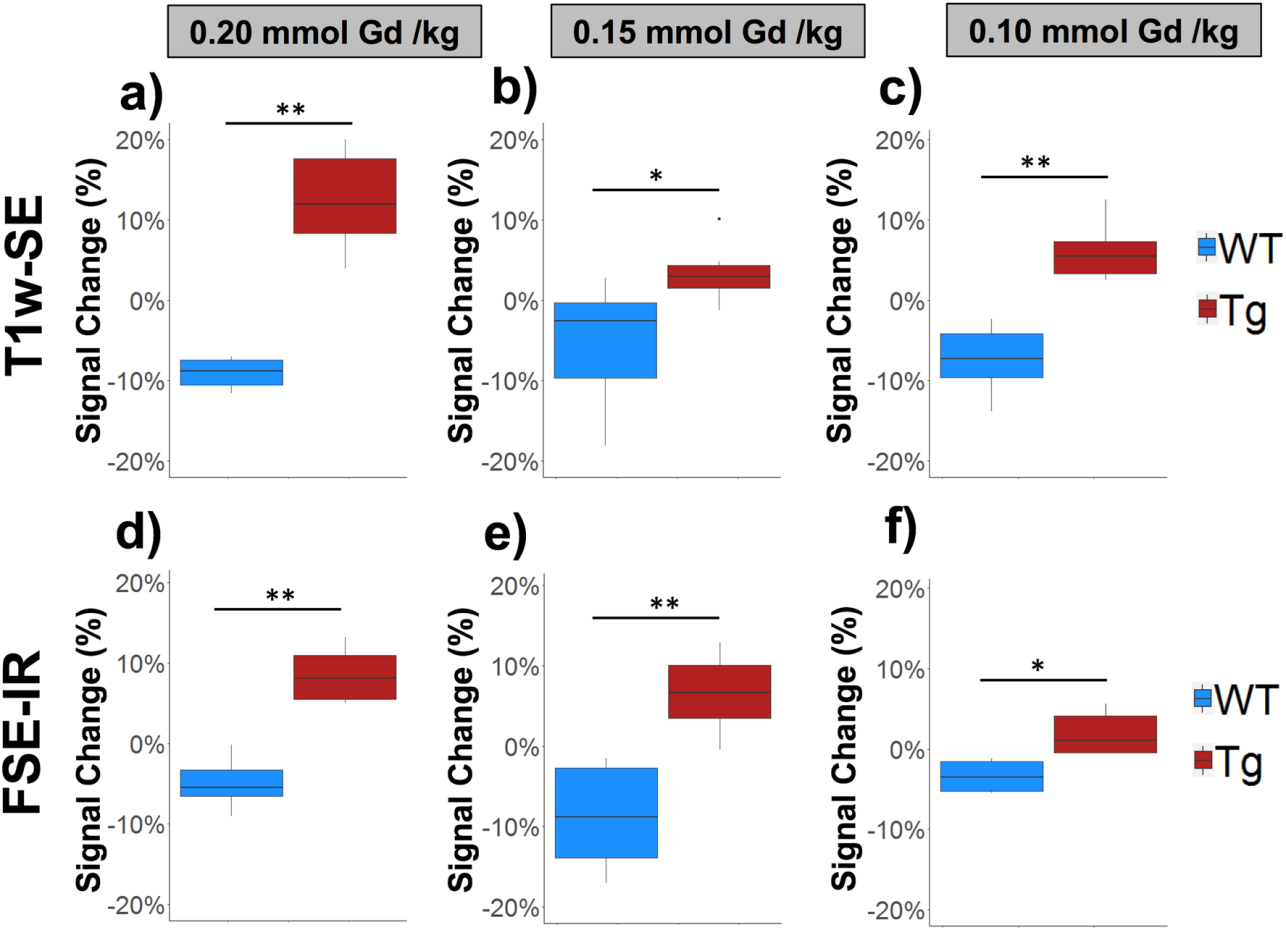
Transgenic (Tg) APPswe/PSEN1dE9 mice demonstrate MR signal enhancement in cortical brain regions relative to wild type (WT) counterparts at all dose levels of **ADx-001**. Box plots show signal changes (expressed as percentage) between pre-contrast and delayed post-contrast T1-weighted spin-echo (T1w-SE) images for (**a**) 0.20 mmol Gd/kg, (**b**) 0.15 mmol Gd/kg, and (**c**) 0.10 mmol Gd/kg dose levels of **ADx-001**. Similar signal changes are shown between pre-contrast and delayed post-contrast fast spin-echo inversion recovery (FSE-IR) images at (**d**) 0.20 mmol Gd/kg, (**e**) 0.15 mmol Gd/kg, and (**f**) 0.10 mmol Gd/kg dose levels. Six mice were tested for both genotypes (Tg and WT) at all three dose levels (0.20, 0.15, and 0.10 mmol Gd/kg) for a total of 36 mice. Wilcoxon rank-sum test: p < 0.05 (*) and p < 0.005 (**).

MRI was performed using a T1-weighted spin echo (T1w-SE) sequence and a fast spin echo inversion recovery (FSE-IR) sequence in transgenic APPswe/PSEN1dE9 mice and age-matched wild type counterparts at three dose levels of **ADx-001**: 0.20, 0.15, and 0.10 mmol Gd/kg. Wild type mice (amyloid-negative) demonstrated little to no brain signal enhancement in delayed post-contrast images (4 days after IV administration) acquired using T1w-SE or FSE-IR at any dose level of **ADx-001**. However, transgenic mice (amyloid-positive) demonstrated high MR signal enhancement in the cortical and hippocampal regions in T1w-SE delayed post-contrast images at **ADx-001** dose of 0.20 mmol Gd/kg (Fig. 2). At 0.15 and 0.10 mmol Gd/kg dose levels, T1w-SE images demonstrated mild signal enhancement in the cortex. Transgenic mice demonstrated moderate to high signal enhancement in delayed post-contrast FSE-IR images at **ADx-001** doses of 0.20 and 0.15 mmol Gd/kg, and mild signal enhancement at 0.10 mmol Gd/kg (Fig. 3). Quantitative analysis of cortical ROIs confirmed qualitative observations of MR signal enhancement in post-contrast delayed images and found a statistically significant difference between transgenic and wild type mice at all dose levels (Fig. 4).

Sensitivity and specificity analysis were performed against gold standard post-mortem brain amyloid staining. A signal variance threshold was estimated from pre-contrast (baseline) scans of all tested mice after establishing that the pre-contrast signal for wild type and transgenic mice was indistinguishable (see Supplementary Fig. 1 online). Estimated baseline signal thresholds were: 5.1% (FSE-IR) and 5.6% (T1w-SE). Amyloid-positive mice were identified if they demonstrated signal enhancement above these cutoffs. Using these thresholds, **ADx-001** demonstrated excellent specificity (100%) at all dose levels using both T1w-SE and FSE-IR sequences (Table 1). In T1w-SE imaging and FSE-IR imaging, **ADx-001** demonstrated high sensitivity (> 80%) at 0.20 mmol Gd/kg dose level. T1w-SE did not demonstrate > 50% sensitivity at lower dose levels (0.15 and 0.10 mmol Gd/kg), while FSE-IR demonstrated 66.7% sensitivity (4/6 true positives identified) at 0.15 mmol Gd/kg. Longitudinal imaging studies in wild type and transgenic mice demonstrated that signal enhancement was optimal four days post-contrast administration and that signal had returned to near-baseline levels by 21 days post-contrast administration (see Supplementary Fig. 2 online).

**Table 1:** In vivo performance for **ADx-001** contrast-enhanced MRI for identification of amyloid positive mice at different dose levels of **ADx-001**.

**ADx-001** dose (mmol Gd/kg)	T1w-SE			FSE-IR		
	Accuracy (%)	Sensitivity (%)	Specificity (%)	Accuracy (%)	Sensitivity (%)	Specificity (%)
0.20	91.7	83.3	100	91.7	83.3	100
0.15	58.3	16.7	100	83.3	66.7	100
0.10	75	50	100	58.3	16.7	100

Six mice were tested for both genotypes (Tg and WT) at all three dose levels (0.20, 0.15, and 0.10 mmol Gd/kg) for a total of 36 mice.

**Figure 5:**
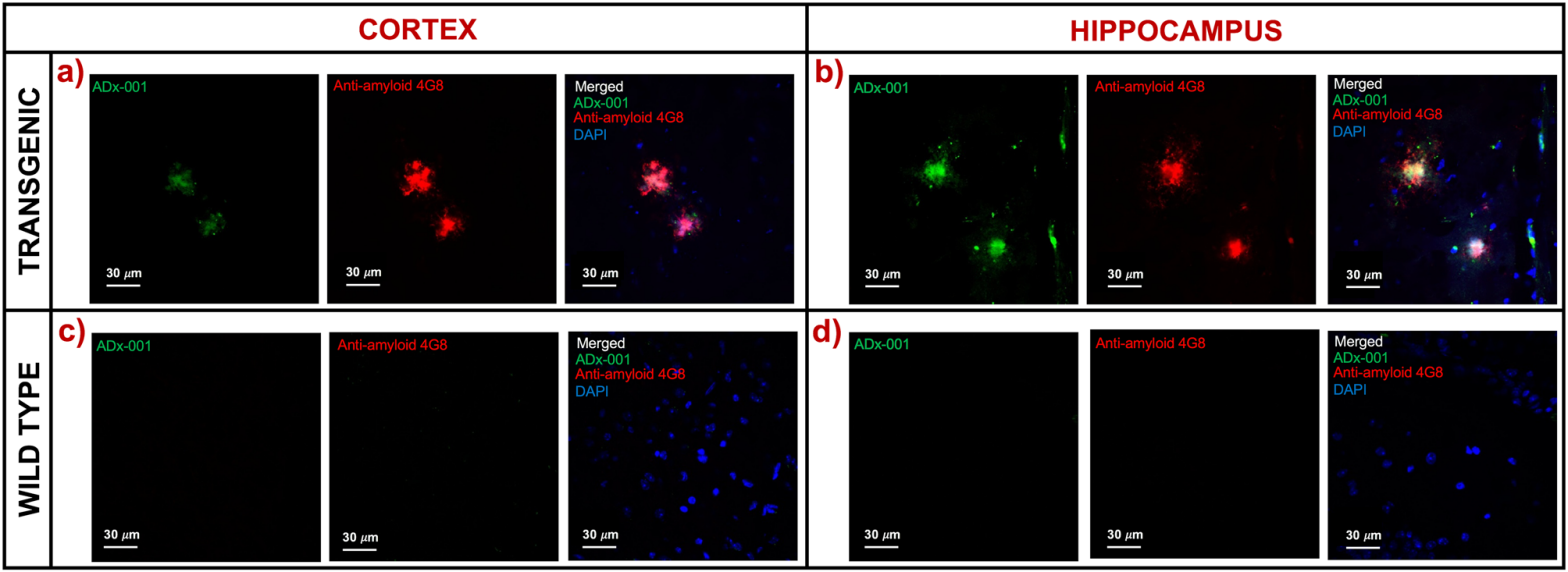
Post-mortem confirmation of **ADx-001** binding to β amyloid plaques. Representative fluorescence microscopy images of **ADx-001** binding to amyloid plaques in (**a**) cortex and (**b**) hippocampus regions in a transgenic mouse. Representative images are also shown for wild type (**c**) cortex and (**d**) hippocampus regions. Wild type mice did not show evidence of amyloid plaque deposits (4G8 antibody staining) or presence of bound **ADx-001** (observing for amyloid ligand fluorescence signal). Images were acquired at 60 × magnification.

Immunofluorescence microscopy analysis confirmed preferential concentration and co-localization of **ADx-001** with amyloid plaque deposits in cortex and hippocampus regions in transgenic mice (Fig. 5). In comparison, wild type mice did not demonstrate amyloid plaque deposits, nor did they show presence of bound **ADx-001** nanoparticles.

### Biodistribution and pharmacokinetics

**Figure 6:**
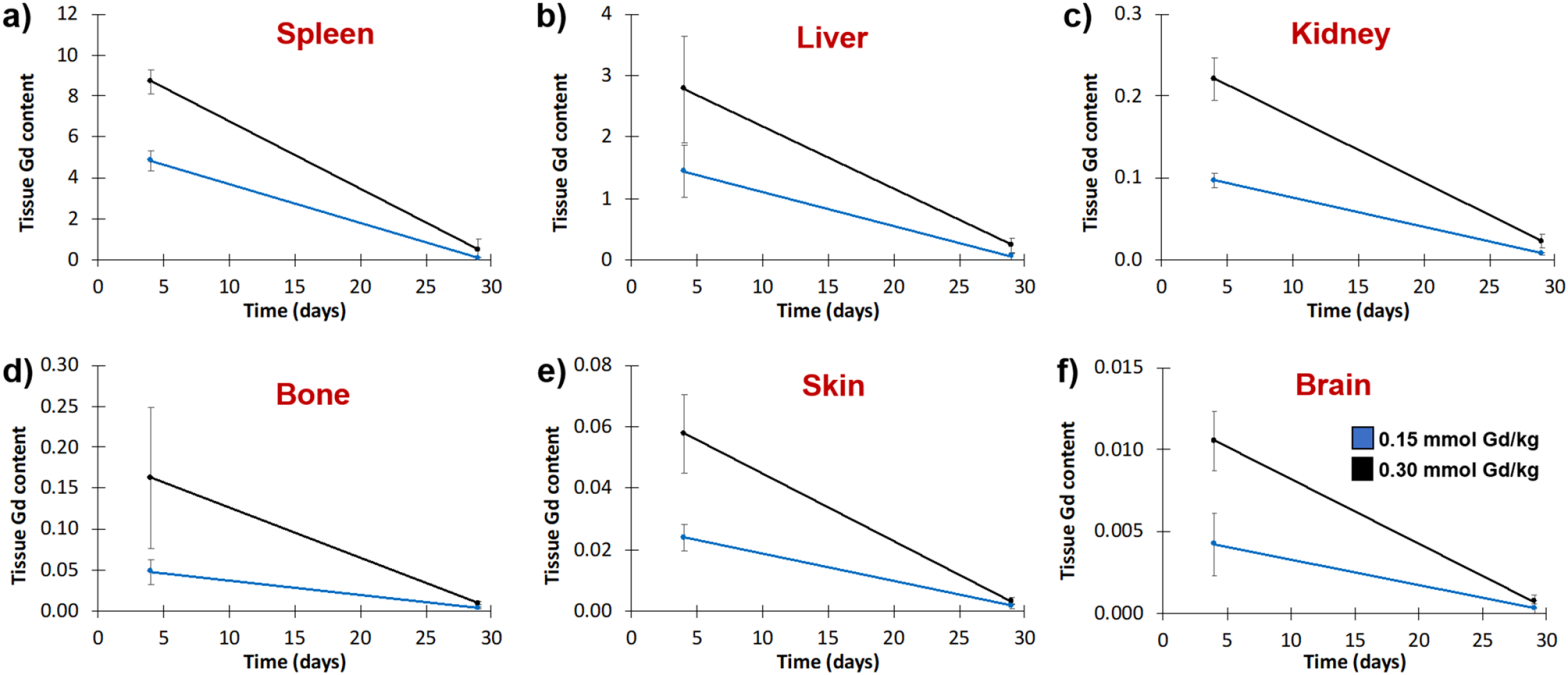
Biodistribution of **ADx-001** in a rat model. Gadolinium levels in (**A**) spleen (**B**) liver (**C**) kidney (**D**) bone (**E**) skin, and (**F**) brain at day 4 and day 28 after intravenous administration of **ADx-001** at 0.15 mmol Gd/kg dose level (blue lines) and 0.3 mmol Gd/kg dose level (black lines). Tissue Gd content is expressed as μmol Gd per gram of wet tissue.

**ADx-001** was well tolerated in rats at doses up to 0.30 mmol Gd/kg. All rats survived until the terminal necropsy at 28 days post-treatment without clinical signs of physiologic dysfunction or physical impairment, with no evidence of adverse effects on clinical signs of toxicity, body weights, clinical pathology, and gross or microscopic changes in examined tissues (data not shown). The no-observed-adverse-effect-level (NOAEL) was 0.30 mmol Gd/kg, the highest dose tested. Tissue Gd levels showed a dose-related increase in all organs (Fig. 6). The highest Gd tissue levels were observed in liver and spleen, which are known to be major organs of clearance of PEGylated liposomal agents^19^. The lowest Gd levels were observed in the skin and brain. Tissue levels of Gd at day 28 were reduced by more than 90% compared to Gd tissue levels at day 4 in all organs.

**Figure 7:**
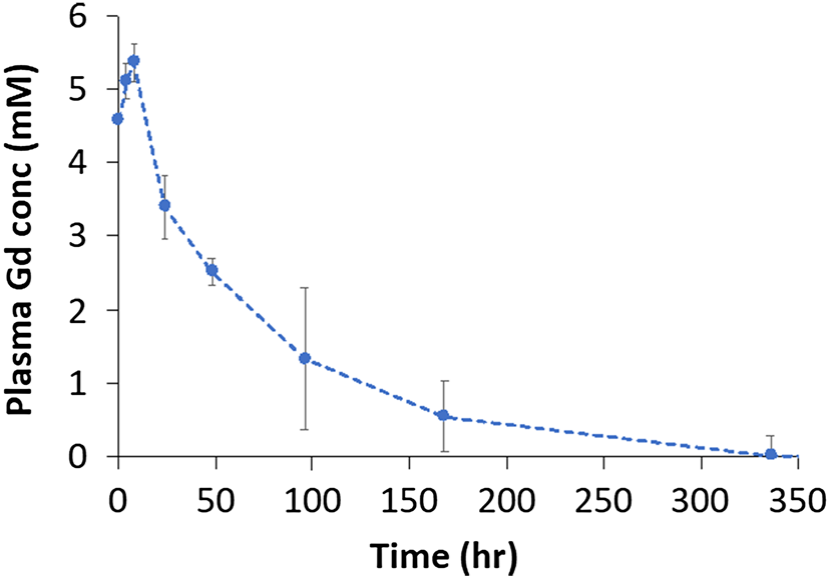
Pharmacokinetic analysis in cynomolgus monkeys demonstrates the long plasma half-life of **ADx-001.** Plasma gadolinium (Gd) concentration were determined using inductively-coupled plasma mass spectrometry (ICP-MS) at various time points after administration of **ADx-001** in cynomolgus monkeys.

Single IV administration of **ADx-001** was also well tolerated in cynomolgus monkeys with no adverse effects seen at dose levels up to 0.30 mmol Gd/kg, the NOAEL. All animals survived the 28-day duration of the study, and no adverse effects were seen on clinical signs of toxicity, clinical pathology or body weights; necropsies were not performed and animals were returned to the laboratory colony at the end of the study. The Gd plasma elimination curves exhibited an alpha and beta phase, but an insufficient number of sampling time points prevented the calculation of elimination rate in the beta phase (Fig. 7). However, assuming first-order kinetics, the elimination rate was 0.017 h^−1^, resulting in a plasma half-life of approximately 41 h (hour). Plasma levels of Gd declined by ~ 80% at 96 h post-start of infusion (SOI) and by greater than 99% at 336 h post-SOI.

## Discussion

Current imaging agents for in vivo detection of AD biomarkers are based on radioisotope PET tracers^20^. Although these agents have contributed to understanding of pathogenesis in Alzheimer’s disease, they pose significant challenges for use in clinical practice due to the high cost PET exams and the limited availability for patients living in rural and remote regions. MRI is one of the most commonly available and utilized neuroimaging modalities with over five times the number of installed instruments as PET machines in the US (even more in other countries), costs less (~ 25% of a PET scan) and is already routinely used in AD patient workup^21–25^. A contrast agent for MRI-based detection of AD biomarkers could therefore have immense clinical utility. Nanoparticles are particularly suited for the development of molecular imaging MR contrast agents due to their ability to carry a high payload of imaging moieties^26^. It has been shown that liposomal nanoparticles presenting gadolinium chelates on their surface exhibit superior T1 relaxivity compared to conventional small molecule Gd contrast agents^27^. Due to high particle-based T1 relaxivity, these agents could provide sensitive read-out of in vivo molecular targets. Furthermore, unlike conventional Gd-based contrast agents, liposomal-Gd agents exhibit superior performance at low, clinically relevant field strengths. The liposomal-Gd platform, containing Gd-DTPA imaging moieties, has been utilized for the development of an amyloid-targeted MR contrast agent for in vivo MR imaging of amyloid plaque deposits in mouse models of AD^17^. However, due to emerging evidence of poor in vivo stability of linear Gd chelates, a novel amyloid-targeted liposomal-Gd contrast agent (**ADx-001**) was developed based on highly stable macrocyclic Gd-DOTA imaging moiety. Additionally, liposomal Gd-DOTA, with Gd-DOTA conjugated to a phospholipid, exhibits ~ three-fold higher T1 relaxivity (~ 31 mM^−1^ s^−1^ on a Gd-basis and ~ 2,295,000 mM^−1^ s^−1^ on a nanoparticle-basis at 1 T field strength) than liposomal Gd-DTPA (~ 9.0 mM^−1^ s^−1^ on a Gd-basis and ~ 668,000 mM^−1^ s^−1^ on a nanoparticle-basis at 1 T field strength) where Gd-DTPA is conjugated to bis(stearylamide)^27–29^. The targeting molecule in **ADx-001** (Fig. 1) is a novel DSPE-PEG3500-styryl-pyrimidine previously shown to strongly bind amyloid plaques when incorporated into liposomes, with a binding constant ~ 3.3 µM^17^.

**ADx-001** was well tolerated in rats and monkeys with the NOAEL in both species being the highest dose tested, 0.30 mmol Gd/kg. All animals survived the studies with no evidence of clinical toxicity or adverse effects on body weight or clinical pathology endpoints (data not shown). Rat tissues evaluated for histopathological changes also showed no evidence of toxicity (data not shown). Pharmacokinetic studies in monkeys showed that **ADx-001** has a long plasma circulation half-life (~ 41 h). While there is a lack of literature on blood half-life of comparable liposome-based MRI contrast agents in monkeys, studies in mice have shown blood half-life in the 14–24 h range^30,31^. Biodistribution studies in rats suggested that the systemic clearance of **ADx-001** is primarily via the liver and spleen, organs of the reticuloendothelial system, consistent with previous reports for liposomes and other nanoparticle agents e.g. Doxil^®^^19^. Tissue levels in rats were reduced by > 90% between Days 4 and 28, suggesting on-going clearance. There was no evidence of substantial long-term retention of Gd in any of the tissues examined and no evidence of **ADx-001**-related toxicity in these organs despite the relatively slow clearance of Gd.

Dose-ranging in vivo efficacy studies were performed in double transgenic APP/Psen1 mice that exhibit progressive build-up of β amyloid brain deposits. In order to investigate translational feasibility, imaging studies were performed on a clinically relevant 1 T MR field strength scanner. Two MR sequences were evaluated for **ADx-001**—enhanced amyloid imaging: (1) a T1w-SE sequence and (2) a modified FSE-IR sequence to approximate a T1w-FLAIR sequence. The FSE-IR sequence was tested due to its high sensitivity to T1-relaxation agents^32,33^. Neither sequence showed MR signal enhancement in control wild type mice above the estimated signal variance thresholds at any **ADx-001** dose level. The absence of amyloid deposits in wild type mice was confirmed by post-mortem immunofluorescence microscopy. A control, non-targeted contrast agent was not included in the current study since previous work demonstrated that such an agent does not cause MR signal enhancement in amyloid-positive mice and does not show non-specific binding to amyloid plaques as confirmed by fluorescence microscopy^17^.

**ADx-001**-enhanced MRI with both sequences demonstrated very high specificity (100%) at all dose levels. Despite all dose levels demonstrating statistically significant signal increase post-contrast administration, several of the transgenic mice at the lower dose levels did not show signal enhancement above the baseline thresholds calculated from pre-contrast scan variance. **ADx-001**-enhanced MRI using an FSE-IR sequence at a dose level of 0.20 mmol Gd/kg demonstrated high sensitivity (> 80%) for identifying amyloid-positive transgenic mice. Delayed post-contrast images acquired four days after administration of **ADx-001** demonstrated signal enhancement in cortex and hippocampus regions. These findings are consistent with known locations of amyloid pathology in APP/Psen1 homozygous mice^34^. **ADx-001**-enhanced MRI using a T1w-SE sequence also demonstrated high sensitivity (> 80%) at 0.20 mmol Gd/kg dose level, but not at 0.15 mmol Gd/kg (16.7%) or 0.10 mmol Gd/kg (50%). The FSE-IR sequence demonstrated high sensitivity at 0.20 mmol Gd/kg, moderate sensitivity at 0.15 mmol Gd/kg (66.7%), and low sensitivity at 0.10 mmol Gd/kg (16.7%). A decrease in sensitivity at lower **ADx-001** dose levels along with statistically significant post-contrast signal enhancement in transgenic mice suggests that further improvements in imaging sequence parameters could improve the accuracy of **ADx-001** for the detection amyloid plaques. The results from this study are consistent with a previous study that reported high accuracy of contrast-enhanced T1w-SE imaging using an amyloid targeting contrast agent at 0.20 mmol Gd/kg dose level^17^.

Efficacy studies were conducted in the dose range of 0.1–0.2 mmol Gd/kg to encompass doses typical of most conventional Gd-based contrast agents. However, the higher sensitivity in T1w-SE imaging at 0.10 mmol Gd/kg relative to 0.15 mmol Gd/kg warrants further investigation at lower dose levels of **ADx-001**.

An amyloid-targeted liposomal macrocyclic gadolinium contrast agent was well tolerated and demonstrated high sensitivity and excellent specificity for in vivo MRI of amyloid plaques. The availability of an MRI-based amyloid imaging agent would have immense utility in clinical research and in studies monitoring novel disease-modifying therapies.

## Materials and methods

All methods were carried out in accordance with relevant guidelines and regulations.

### ADx-001

**ADx-001** was provided as a sterile, ready-to-use injectable liquid (Alzeca Biosciences, Houston, USA). **ADx-001** is comprised of PEGylated liposomes in a 150 mM NaCl/10 mM Histidine buffer. The liposomes incorporate Gd(III)-DSPE-DOTA (the macrocyclic gadolinium imaging moiety, Gd(III)-DOTA, conjugated to a phospholipid, 1,2-Distearoyl-sn-glycero-3-phosphorylethanolamine, DSPE) and **ET3-73** (amyloid plaque targeting moiety, displaying a styryl-pyrimidine terminal on DSPE-PEG-3500,1,2-distearoyl-sn-glycero-3-phosphoethanolamine-N-[maleimide(polyethylene glycol)-3500]) in the bilayer (Fig. 1). The overall Gd concentration is 25 mM. Liposome particle size measured by dynamic light scattering is 140 nm. Zeta potential was ~ − 38 mV, consistent with PEGylated liposomes^35^.

### Animal studies

Mouse imaging studies were performed under a protocol approved by the Institutional Animal Care and Use Committee (IACUC) of Baylor College of Medicine. Rodent and monkey toxicity, pharmacokinetic and tissue distribution studies were performed under protocols approved by the IACUC of Charles River Laboratories, Reno, NV. At the end of studies, mice and rats were euthanized as per the recommendations of the American Veterinary Medical Association (AVMA) Guidelines on Euthanasia. Rodents were euthanized by CO_2_ inhalation (primary) and cervical dislocation (secondary). Monkeys were not euthanized and were returned to the laboratory animal colony at the end of the study.

### Magnetic resonance imaging (MRI) study

Imaging studies were performed in an APPswe/PSEN1dE9 (C57BL/6 J background, 11–18 months age) double transgenic mouse model of early-onset Alzheimer’s disease (JAX MMRRC Stock #005864, Jackson Laboratory, Bar Harbor, ME). The transgenic mice develop amyloid plaques in the brain around 6–7 months of age. **ADx-001** was tested at three dose levels (mmol Gd/kg): 0.10, 0.15 and 0.20. At each dose level, **ADx-001** was tested in transgenic (n = 6) and age-matched wild type (mice that lacked both mutations) mice (n = 6). Mice were restrained and **ADx-001** was intravenously administered via tail vein injection.

Magnetic Resonance Imaging (MRI) was performed on a 1 T permanent magnet scanner (M7 system, Aspect Imaging, Shoham, Israel) using methods previously described by our group^17,29^. Mice were sedated using 2.5% isoflurane and then placed on a custom fabricated bed with an integrated face-cone for continuous anesthesia delivery by inhalation (1–2% isoflurane). Respiration rate was monitored by a pneumatically controlled pressure pad placed underneath the abdomen of the mice. Two MRI sequences were tested: a T1-weighted spin-echo (T1w-SE) sequence and a 2D fast spin echo inversion recovery (FSE-IR) that approximates a fluid-attenuated inversion recovery (FLAIR) sequence. SE parameters: TR = 600 ms, TE = 11.5 ms, slice thickness = 1.2 mm, matrix = 192 × 192, FOV = 30 mm, slices = 16, NEX = 4. FSE-IR parameters: TR = 6500 ms, TE = 80 ms, TI = 2000 ms, slice thickness = 2.4 mm, matrix = 192 × 192, FOV = 30 mm, slices = 6, NEX = 6. Coil calibration, RF calibration, and shimming were performed at the beginning of study for each subject. All mice underwent pre-contrast scans followed by intravenous administration of **ADx-001**. Delayed post-contrast scans were acquired four days after administration of contrast agent. Pre-contrast and post-contrast scans were acquired using both T1w-SE and FSE-IR sequences with the parameters listed above.

To account for variability between mice and potential artifacts due to positioning or MR instrument factors, we determined the mean and standard deviation for MR signal intensity for all wild type and transgenic mice for both the T1w-SE and FSE-IR sequences (see Supplementary Fig. 1 online). A cutoff threshold signal intensity, set as two standard deviations above the mean, was then estimated for both sequences and represented as a percentage of mean signal intensity: 5.1% (FSE-IR) and 5.6% (T1w-SE).

A longitudinal study of transgenic (n = 3) and wild type mice (n = 3) was performed to validate the peak post-contrast signal (Supplementary Fig. 2 online). All six animals underwent pre-contrast scans and were injected with **ADx-001** (0.20 mmol Gd/kg) via tail vein injection. Animals were then imaged at 2, 4, 6, 8, and 21 days post-contrast administration. This study determined that the optimal time point for capturing signal enhancement was 4 days post-contrast administration. Signal enhancement levels returned to baseline levels between 8 and 21 days post-contrast administration.

Wild type and transgenic mice were euthanized after post-contrast scans and perfused with 0.9% saline followed by 4% formalin solution. The brains were excised and fixed in 4% formalin solution for 24 h and then transferred to 30% sucrose for cryoprotection. Brains were embedded in OCT and stored at − 80 °C until ready for sectioning. Brain sections (15 µm thick) were cut and used for post-mortem phenotypic confirmation of amyloid deposition. Sections were incubated in 5% Bovine Serum Albumin (BSA) for 1 h, followed by incubation with fluorescent-tagged anti-amyloid β antibody (AF647-4G8, BioLegend, San Diego, CA) in 3% BSA at 4 °C overnight. Sections were further stained with a nuclear marker (DAPI), washed, mounted, cover-slipped using Vectashield mounting medium (Vector Laboratories, Burlingame CA), and imaged on a confocal microscope with appropriate filter sets. The presence of amyloid bound **ADx-001** nanoparticles was analyzed by fluorescence imaging for the amyloid targeting ligand (Ex/Em: 406 nm/460 nm).

Qualitative and quantitative analysis of MRI images was performed in OsiriX (version 5.8.5, 64-bit, Pixmeo SARL, Geneva, Switzerland) and MATLAB (version 2015a, MathWorks, Natick, MA). Brain extraction was performed through a combination of threshold and manual segmentation in OsiriX. Signal change between pre-contrast and delayed post-contrast images was assessed through quantification of signal intensity (SI) in cortical regions near the center of the image stack (see Supplemental Fig. S3 online). Amyloid-positive mice were identified through assessment of signal enhancement between pre-contrast and delayed post-contrast assessment of the cortex. The change in signal between pre-contrast and post-contrast images was quantified through integration of signal in regions of interest (ROI) that encompassed cortical tissue in central slices of the MRI volume. Signal change (%) was calculated as in Eq. (1).

1 $$ {\text{Signal }}\,\,{\text{change}} \left( \% \right) = 100 \times \frac{{SI_{Post} - SI_{Pre} }}{{SI_{PRE} }} $$

Post-mortem brain amyloid staining using the 4G8 antibody was used as the gold standard for the determination of true positives and true negatives. An observation of signal enhancement in **ADx-001**-enhanced delayed MR images of an amyloid-positive mouse (as determined by immunofluorescence) above the signal variance threshold was counted as a true positive result. Conversely, signal enhancement below the signal variance threshold between pre-contrast and delayed post-contrast images for an amyloid-negative mouse was considered a true negative. Additionally, a false positive was defined as signal enhancement in an amyloid-negative mouse whereas a false negative was defined as absence of signal enhancement in an amyloid-positive mouse. Sensitivity was determined by the ratio of MRI-identified true positives to the sum of true positives and false negatives. Specificity was determined as the ratio of MRI-identified true negatives to the sum of true negatives and false positives. Overall accuracy was calculated as the ratio of number of mice correctly identified by MRI to the total number of mice studied.

### Tissue biodistribution

The biodistribution of **ADx-001** was studied in Wistar Han rats. Male rats (10 weeks age, 257–296 g body weight; n = 13 per treatment group; Charles River Laboratories, Kingston, NY) were restrained and administered **ADx-001** at 0, 0.15 or 0.30 mmol Gd/kg as a single intravenous bolus injection. Standard toxicology endpoints were included in this study, including clinical toxicity, body weights, clinical pathology (clinical chemistry, hematology and coagulation) and macro- and microscopic pathology. Animals were euthanized at Day 4 (n = 7/dose level) or Day 28 (n = 6/dose level) post-administration of **ADx-001** for complete necropsies. Selected tissues (bone, brain, kidney, liver, skin and bone) were collected for histopathological analysis and determination of Gd levels. For determination of Gd levels, tissue samples were frozen immediately in liquid nitrogen and stored at − 20 °C until analysis.

Gd concentration in tissue samples was quantified using inductively-coupled plasma mass spectrometry (ICP-MS) (Agilent, CA, USA). Wet tissue (~ 100 mg) was digested in 90% concentrated HNO_3_ (~ 750 μL) at 90 °C for 10–15 min. The digested sample was diluted in deionized (DI) water, vortexed vigorously and centrifuged at 3500 rpm for 15 min. The supernatant was separated and further diluted as needed to ensure Gd concentrations fell within the range of calibration standards (1–500 ppb). Quality control samples (50 and 100 ppb) were included at the start, middle and end of analysis runs.

### Pharmacokinetic studies

The pharmacokinetics (PK) of **ADx-001** were evaluated in cynomolgus monkeys. Briefly, non-naïve male cynomolgus monkeys (n = 3 per treatment group, 2–5 yr age, 2.3–3.1 kg body weight; Charles River Laboratories, Reno, NV) were chair-restrained and intravenously administered **ADx-001** using a calibrated infusion pump over ~ 60 min at 0.30 mmol Gd/kg. Blood samples were collected from all animals pre-dose, immediately post-end of infusion, and 4, 8, 24, 48, 96, 168, 336, and 672 h post-start of infusion (SOI). Blood samples were processed to plasma and stored frozen until ready for analysis. This was a non-necropsy study and the assessment of **ADx-001** toxicity was limited to clinical toxicity, body weights and clinical pathology (clinical chemistry, hematology and coagulation) measurements. Animals were returned the laboratory colony at the termination of the study.

Nanoparticle-based products are known to elicit infusion reactions via the complement pathways and are commonly known as complement activation-related pseudoallergy (CARPA)^36^. Certain large animal species, including monkeys, may be hypersensitive to nanoparticle administration. However, studies have shown that administration of nanoparticles via a slow infusion mitigates CARPA. As a result, **ADx-001** was administered as a slow infusion in monkeys. Rodents generally do not exhibit this reaction and can sustain bolus injections within the volumetric limits of infusion.

Gd concentration in plasma samples was determined using ICP-MS (Agilent, CA, USA). Plasma samples (100 μL) were digested in 90% concentrated HNO_3_ (750 μL) at 90 °C for 15 min. The digested samples were diluted in deionized (DI) water, centrifuged at 3000 rpm for 15 min and the supernatant was further diluted for ICP-MS analysis such that the Gd concentrations fell within the range of ICP-MS calibration standards (1–500 ppb).

### Statistical analysis

A Wilcoxon rank-sum statistical test was applied to compare group differences. Values of p ≤ 0.05 were considered statistically significant.

## Supplementary information

Supplementary file 1

## Data Availability

Data supporting the findings of this study are available from the corresponding author on request.
