# Supplementary material for: Pre-clinical dose-ranging efficacy, pharmacokinetics, tissue biodistribution, and toxicity of a targeted contrast agent for MRI of amyloid deposition in Alzheimer’s disease
Source: Sci Rep. 2020 Sep 30;10:16185. doi: 10.1038/s41598-020-73233-7 (PMC7527957; doi:10.1038/s41598-020-73233-7)
Supplement: Supplementary file 1 [file 41598_2020_73233_MOESM1_ESM.pdf]

Supplementary Information

Pre-clinical dose-ranging efficacy, pharmacokinetics, tissue biodistribution, and toxicity of a targeted contrast agent for MRI of amyloid deposition in Alzheimer's disease

Authors and affiliations:

Andrew A. Badachhape, Ph.D., Department of Radiology, Baylor College of Medicine, Houston, TX 77030, USA, badachha@bcm.edu

Peter K. Working, Ph.D., Alzeca Biosciences Inc., Houston, TX 77057, USA, peterworking@workingadvisors.com

Mayank Srivastava, Ph.D., The Singleton Department of Pediatric Radiology, Texas Children's Hospital, Houston, TX 77030, USA, mxsrivas@texaschildrens.org

Prajwal Bhandari, M.S., Department of Radiology, Baylor College of Medicine, Houston, TX 77030, USA, prajwal.bhandari@bcm.ede

Igor V. Stupin, Ph.D., The Singleton Department of Pediatric Radiology, Texas Children's Hospital, Houston, TX 77030, USA, ivstupin@texaschildrens.org

Laxman Devkota, Ph.D., Department of Pediatrics-Oncology, Baylor College of Medicine, Houston, TX 77030, USA, laxman.devkota@bcm.edu

Eric A. Tanifum, Ph.D., The Singleton Department of Pediatric Radiology, Texas Children's Hospital, Houston, TX 77030, USA, eatanifu@texaschildrens.org

Ananth V. Annapragada, Ph.D., The Singleton Department of Pediatric Radiology, Texas Children's Hospital, Houston, TX 77030, USA, avannapr@texaschildrens.org

*Ketan B. Ghaghada, Ph.D., The Singleton Department of Pediatric Radiology, Texas Children's Hospital, Houston, TX 77030, USA, kbghagha@texaschildrens.org

***Corresponding author:**

Ketan B. Ghaghada, Ph.D.
Edward B. Singleton Department of Radiology, Texas Children's Hospital
Department of Radiology, Baylor College of Medicine
1102 Bates Street, Suite 850
Houston, TX 77030
Phone: 832-824-0861
Email: kbghagha@texaschildrens.org

Supplementary Information

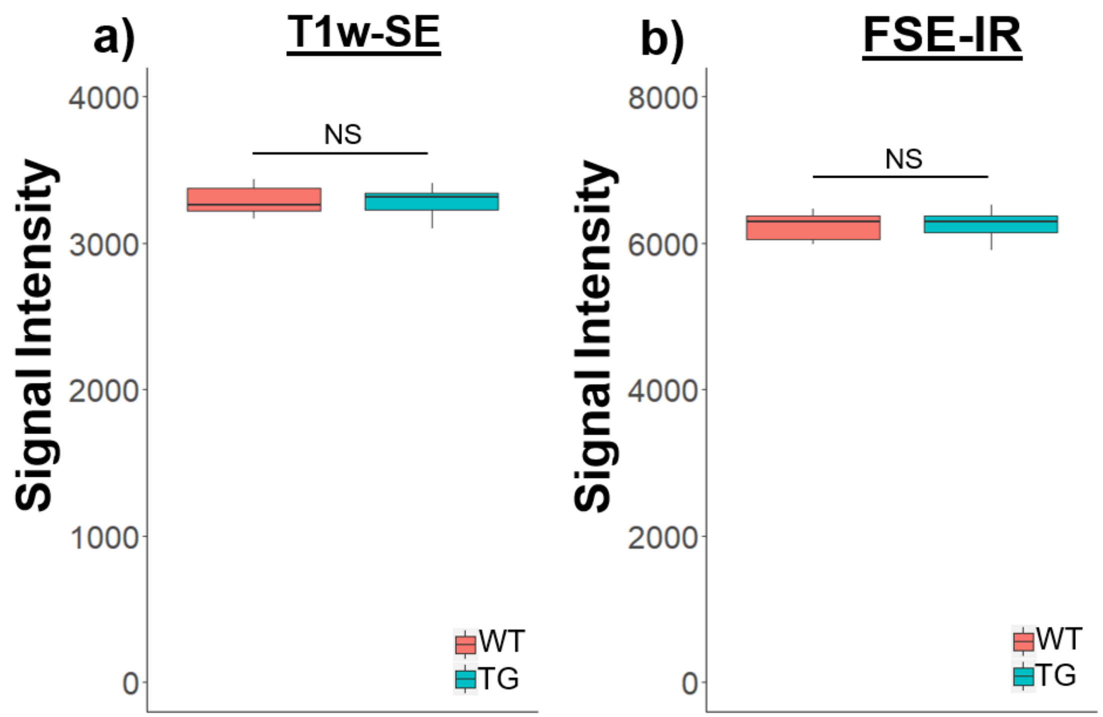

Supplementary Figure S1: No significant differences in signal intensity were seen in pre-contrast magnetic resonance imaging (MRI) scans of transgenic and wild type mice. Signal intensity mean and range for **(a)** T1-weighted spin echo (T1w-SE), and **(b)** fast spin echo inversion recovery (FSE-IR) sequences for pre-contrast scans of both wild type (n=18) and transgenic (n=18) animals. Regions of interest (ROI) were drawn in the cortex for each animal. No significant differences (NS) were found between signal intensities for wild type and transgenic animals for either T1w-SE or FSE-IR sequences.

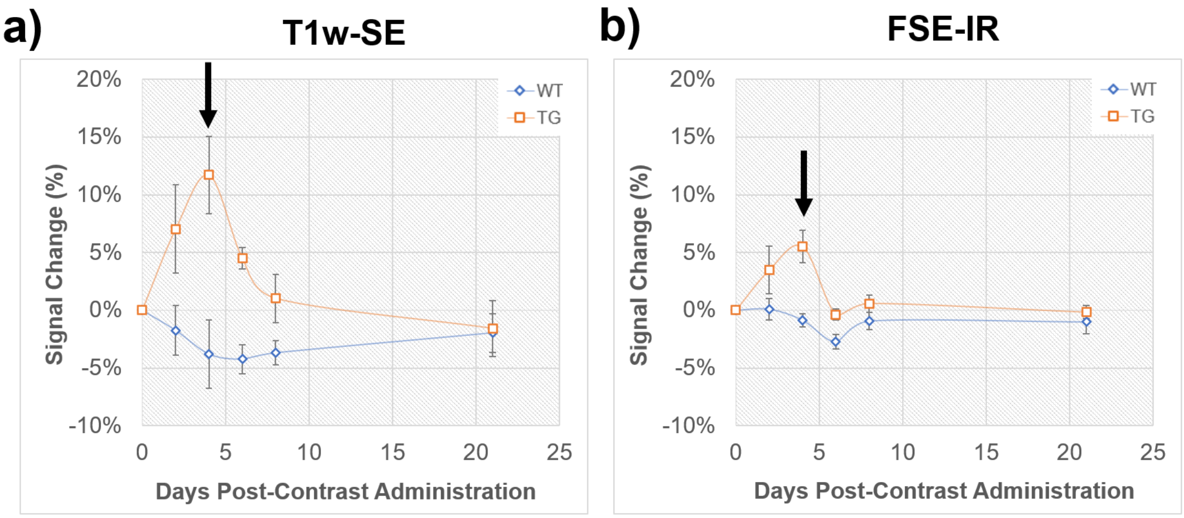

Supplementary Figure S2: Determination of optimal time point for imaging of amyloid plaques. Three transgenic and three wild type mice were imaged before (Day 0) and after administration of ADx-001 at 2, 4, 6, 8, and 21 days post-contrast. Maximum signal enhancement was seen at day 4 post-contrast (black arrow). Transgenic animals demonstrated signal enhancement relative to wild type animals in both **(a)** T1-weighted spin echo (T1w-SE) and **(b)** fast spin echo inversion recovery (FSE-IR) sequences. Signal returned to near baseline levels by day 21.

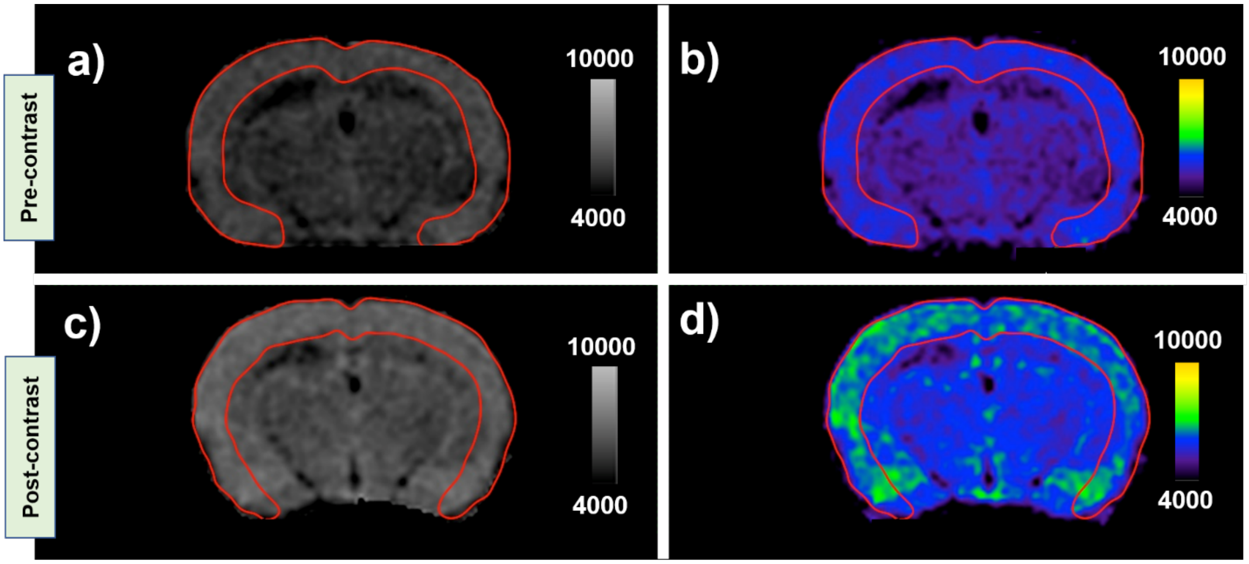

Supplementary Figure S3: Demonstration of cortical regions of interest (ROI) identification. Cortical ROIs are outlined in red on fast spin echo inversion recovery (FSE-IR) brain images. Pre-contrast gray scale **(a)** and post-contrast gray scale **(c)** images are compared with pre-contrast **(b)** and post-contrast **(d)** color magnitude images.
